# Intention-based predictive information modulates auditory deviance processing

**DOI:** 10.3389/fnins.2022.995119

**Published:** 2022-09-28

**Authors:** Andreas Widmann, Erich Schröger

**Affiliations:** ^1^Wilhelm Wundt Institute for Psychology, Leipzig University, Leipzig, Germany; ^2^Leibniz Institute for Neurobiology, Magdeburg, Germany

**Keywords:** prediction, audition, intention, perception, action, predictive coding, mismatch negativity (MMN)

## Abstract

The human brain is highly responsive to (deviant) sounds violating an auditory regularity. Respective brain responses are usually investigated in situations when the sounds were produced by the experimenter. Acknowledging that humans also actively produce sounds, the present event-related potential study tested for differences in the brain responses to deviants that were produced by the listeners by pressing one of two buttons. In one condition, deviants were unpredictable with respect to the button-sound association. In another condition, deviants were predictable with high validity yielding correctly predicted deviants and incorrectly predicted (mispredicted) deviants. Temporal principal component analysis revealed deviant-specific N1 enhancement, mismatch negativity (MMN) and P3a. N1 enhancements were highly similar for each deviant type, indicating that the underlying neural mechanism is not affected by intention-based expectation about the self-produced forthcoming sound. The MMN was abolished for predictable deviants, suggesting that the intention-based prediction for a deviant can overwrite the prediction derived from the auditory regularity (predicting a standard). The P3a was present for each deviant type but was largest for mispredicted deviants. It is argued that the processes underlying P3a not only evaluate the deviant with respect to the fact that it violates an auditory regularity but also with respect to the intended sensorial effect of an action. Overall, our results specify current theories of auditory predictive processing, as they reveal that intention-based predictions exert different effects on different deviance-specific brain responses.

## Introduction

Sounds violating an auditory regularity trigger a cascade of deviance-specific brain responses, even when the auditory stimulation is task-irrelevant (Näätänen, 1990). The underlying mechanisms are in the service of detecting “new,” unexpected, yet potentially relevant information. A phenomenological consequence of this deviance-specific processing can be attentional capture, while a behavioral consequence can be impaired performance in a primary task not related to the deviancy (Parmentier, 2014). Current theories of auditory predictive processing postulate that deviance processing is achieved on the basis of neural models representing detected auditory regularities that generate (implicit) predictions about what to expect next (Grimm and Schröger, 2007; Winkler et al., 2009; Escera et al., 2014; Schröger et al., 2015; Heilbron and Chait, 2018). The huge amount of research on this topic is based on experiments where the experimenter controls the delivery of the sounds. However, listeners are also active agents intentionally producing sounds by themselves. Predictive coding theory postulates that actions induce active inference to minimize sensory prediction errors (Friston and Stephan, 2007; Friston et al., 2010; Brown et al., 2013; Clark, 2013). In other words, active behavior should interact with sensory processing. Indeed, it has been shown that self-produced sounds are compared to the intended (predicted) sensorial consequence of the action (Waszak et al., 2012; Hughes et al., 2013), and auditory regularity-based and intention-based predictive processing of sounds interact (Korka et al., 2019; Darriba et al., 2021). The present event-related potential (ERP) study investigates whether and how deviance-specific processing based on auditory regularities is modulated for self-produced sounds.

**FIGURE 1:**
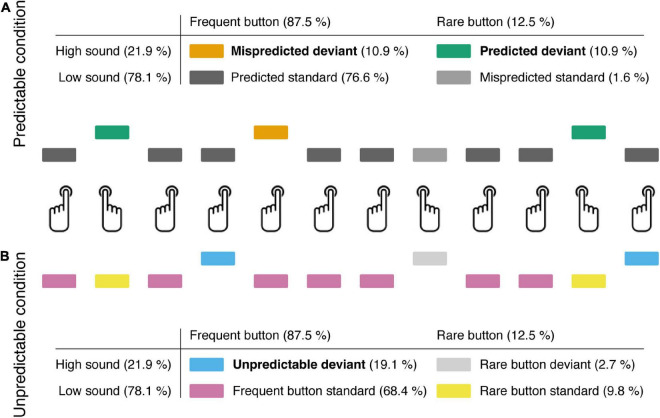
Participants pressed one button frequently and the other button rarely. Button presses generated a frequent low pitch (“standard”) or a rare high pitch (“deviant”) tone. **(A)** In the predictable condition, participants were instructed to generate standard and deviant sounds via the respective buttons. In addition to self-produced “predicted deviants,” the frequently pressed button occasionally elicited a “mispredicted deviant” (instead of a standard). **(B)** In the unpredictable condition, the type of button-press (frequent, rare) and the type of sound (standard, deviant) were unrelated, so that “unpredictable deviants” were triggered.

Participants were asked to press one button frequently and a second button rarely. In one experimental condition the two buttons were not associated with a particular sound, but standard and deviant sounds were randomly and unpredictably presented irrespective of which of the two buttons was pressed (“unpredictable condition”; see Figure 1). In another condition standard and deviant sounds were predictably associated to the two buttons with high validity (“predictable condition”). One button produced a standard sound, and the other button produced a deviant sound (“predicted deviant”) in most trials. However, there were also some incorrectly predicted deviant sounds produced when the button for a standard sound was pressed (“mispredicted deviant”). The present study considers three major deviance-specific ERP effects, namely, the N1 enhancement, the mismatch negativity (MMN) and the P3a.

The N1 (peaking around 100 ms after sound onset) is often reported to be enhanced for deviants relative to standards. This effect can be explained by standard sound-specific adaptation of the N1 eliciting neurons (Näätänen, 1990). When a deviant is presented, (partly) non-adapted neurons are activated resulting in relative enhancement of the N1. Such an effect on scalp-recorded ERPs can be explained by short-term synaptic depression of neurons in auditory cortex causing a transient weakening of synaptic connections (May, 2021). As this theory does not (explicitly) include top-down influence of intentional action, a modulation of the auditory-regularity based N1 enhancement to deviants is not to be expected. Indeed, Korka et al. (2019) did not find a N1 deviance effect for a deviant sound which violated an intention-based prediction. Similarly, Darriba et al. (2021) found no modulations for the Na and Tb subcomponents of the N1 for violations of an expected action effect. Note, however, that the auditory N1 *per se* can be modulated by top-down effects, for example, it is increased when the sound is attended and decreased when the sound is self-generated (for reviews see, Horvath, 2015; Schröger et al., 2015). Thus, one may possibly expect modulations of the N1 oddball effect by intention when perception and action are as strongly linked as in the present paradigm.

Subsequent to and partially overlapping with the N1, the MMN is elicited by violations of an auditory regularity. MMN is often explained as resulting from a mismatch process comparing the actual sound with a prediction derived from an internal model representing the regularity (Garrido et al., 2009; Winkler and Czigler, 2012). Many studies with externally generated sounds reported that the MMN is not modulated by attentional top-down predictive information (for review see e.g., Sussman et al., 2014). The MMN-system is of special interest for the present study because it can process different predictions concurrently and can generate respective MMN responses to violations of these predictions in parallel (Paavilainen et al., 2001, 2003; Wolff and Schröger, 2001; Pieszek et al., 2013). According to an extension of the “auditory event representation system (AERS)” framework (Korka et al., 2022), it is assumed that sound predictions generated by action intention are functionally equivalent to sound predictions generated by an extracted auditory regularity. This is evidenced by the finding that the violation of an intention-based prediction alone–in the absence of an auditory regularity-based prediction–can elicit MMN (Korka et al., 2019).

Do these MMNs for auditory-regularity violation and action-intention violation interact? In a study by Nittono (2006), the MMNs for self-generated sounds triggered by a button press and externally generated sounds did not differ from each other. As deviants were fully unpredictable in this pioneering study, an additional MMN modulation by action intention was not necessarily to be expected by predictive coding theories. In a study by Waszak and Herwig (2007), where two buttons (instead of one as in the Nittono, 2006 study) were associated with standard and deviant sounds in a training phase (but not during the actual experiment), an effect could have been expected by ideomotor theory stating that the perceptual idea of an action (i.e., its anticipated sensorial effect) initiates the selection and execution of that action (Greenwald, 1970; Hommel et al., 2001; Shin et al., 2010). However, Waszak and Herwig (2007) also did not observe a modulation of MMN depending on whether the sounds were elicited by the button associated with the deviant or the button associated with the standard during the training phase (but a modulation of P3a; see below). In a study by Rinne et al. (2001), self-generated deviants yielded a regular MMN even when they were fully predictable due the deterministic button-sound mapping. This suggests that the intention-based prediction of a deviant has no effect on the auditory-regularity-based MMN. In contrast to the study by Rinne et al. (2001), the present study emphasizes an intention-based action mode and included mispredicted deviants (triggered by the button-press that usually produced a standard), both manipulations presumably promoting the monitoring of action effects. Thus, it appears plausible that the auditory-regularity-based MMN might be attenuated when a deviant is predicted according to intended outcome of an action in this experimental scenario. However, if the present study still yields full-fledged MMN, this would be a strong case for a strictly modular organization of the MMN for the violation of an auditory regularity which cannot be accessed by top-down processing of intentional action.

Darriba et al. (2021) reported an enhancement of the auditory deviance effect in the MMN latency range in response to the violation of a learned sound pattern when the sound additionally violated an intended action effect. This possibly indicates two separate, additive rather than interactive routes of prediction violation. The authors labeled this effect peaking 148 ms after sound onset as an effect on the N1b rather than the MMN. As N1b and MMN share important characteristics in terms of latency and supratemporal generators, and as the MMN has also been proposed to be a subcomponent of the N1 wave (Näätänen and Picton, 1987; May and Tiitinen, 2010), the deviance N1b effect and MMN have possibly not been disentangled here. Anyway, unlike the Rinne et al. (2001) study, the studies by Korka et al. (2019) and by Darriba et al. (2021) show that the violation or confirmation of an intention-based prediction can modulate auditory deviance ERP effects in the MMN time range (and Le Bars et al., 2019 for related N2b).

The MMN is often followed by the P3a, which is regarded as indicating a switch of attention toward the deviant sound (Escera et al., 1998; for review see, Polich, 2007). It is assumed that it not only includes an evaluation of the mere physical difference between deviant and standard, but also an evaluation of the potential significance of the stimulus with respect to the aims of the listener (Winkler and Schröger, 2015). According to Nieuwenhuis et al. (2011), the P3a indicates activity of the locus coeruleus-norepinephrine system elicited by motivationally significant stimuli mobilizing resources for action. An increase of P3a has been reported by Nittono (2006; also see, Nittono and Ullsperger, 2000; Knolle et al., 2019) for self-generated sounds (without a specific button-sound association) compared to externally generated sounds presumably due to unequivocal stimulus timing and voluntary stimulus production enhancing orienting of attention explained with reference to the ideomotor theory (for review see, Hommel et al., 2001). The perceptual representation of the forthcoming stimulus is activated by action intention by means of associative learning. Furthermore, in case of established button-sound relationships, the P3a has been observed even by predicted deviants and enhanced for unpredictable deviants (Waszak and Herwig, 2007; Knolle et al., 2019; Darriba et al., 2021). Darriba et al. (2021) suggested that the P3a results indicate that auditory regularity-based and action intention-based sound predictions coexist simultaneously as independent predictions (i.e., parallel and additive). We expect to replicate this result in our experimental scenario.

## Materials and methods

### Participants

Data from 17 participants were recorded. The data from two participants had to be excluded from analysis due to technical problems during the recording. The mean age of the remaining 15 participants was 23.5 years (range 19–36 years). 14 of the participants were right-handed, one left-handed. Eight of the participants were female, seven male. For three participants, the two experimental conditions were recorded in two sessions on separate days. All of them reported normal hearing and normal or corrected to normal vision. None of the participants had a history of a neurological disease or injury. Participants received either course credit or payment (18 Euros) for their participation in the experiment and gave their written informed consent after the details of the procedure had been explained to them. The experiment was conducted according to the Declaration of Helsinki and the ethical guidelines of The German Psychological Society (“Deutsche Gesellschaft für Psychologie”, DGPs^1^) and complied with all institutional and country-specific legal requirements.

### Procedure

The experiment consisted of two conditions, a predictable and an unpredictable condition, each including 12 blocks of 128 trials. In both conditions participants were instructed to produce sounds by button presses and press one button 112 times (87.5%; frequent button) and another button 16 times per block (12.5%; rare button). Each button press was followed by a sound. In a “predictable” condition the type of button-press (frequent and rare) correctly predicted the type of sound (standard and deviant) in most trials, whereas in an “unpredictable” condition, the type of button press and type of sound were unrelated. In the predictable condition, 98 (87.5%) of the 112 frequent button presses were followed by a low sound (predicted standard) and 14 (12.5%) were followed by a high sound (mispredicted deviant). 14 (87.5%) of the 16 rare button presses were followed by a high sound (predicted deviant) and 2 (12.5%) were followed by a low sound (mispredicted standard). Participants were informed that frequent button presses were usually followed by a low sound and rare button presses usually were followed by a high sound and instructed not to care about rare, unexpected sounds. In the unpredictable condition, 87 or 88 (78.1%) of the frequent button presses were followed by a low sound (frequent standard) and 24 or 25 (21.9%) were followed by a high sound (frequent deviant). 12 or 13 (78.1%) of the 16 rare button presses were followed by a low sound (rare standard) and 3 or 4 (21.9%) followed by a high sound (rare deviant). Participants were informed that button presses were followed by either a low sound with higher probability or a high sound with lower probability irrespective whether the frequent or the rare button was pressed. In total, in both conditions, 100 low sounds (78.1%) and 28 high sounds (21.9%) were presented per block. Trials were pseudo-randomized with the constraint that never two mispredicted deviants in the predictable condition and never two frequent deviants in the unpredictable condition directly succeeded each other. We would like to note that sounds were not fully unpredictable in the unpredictable condition as standard sounds were presented with higher probability than deviant sounds. We chose this terminology to emphasize the contrast between conditions with actions (i.e., button presses) predictably associated with action effects (i.e., sound type) in the “predictable” and unpredictably and therefore independent of action selection in the “unpredictable” condition.

Participants were instructed to distribute the infrequent button presses as randomly as possible across the whole block, to press buttons at a regular interval of 800–900 ms, not to press the rare button two times in a row, and to avoid fixed patterns (e.g., pressing the rare button every fifth time). The number of remaining button presses per button per block and the time between the last two button presses were displayed to the participants on a computer screen. If the interval between the last two button presses was shorter than 600 ms or longer than 1,200 ms, or the participant pressed the rare button two times in a row, or pressed buttons in a fixed pattern (if the number of frequent button presses between two rare button presses was identical three times in a row) a visual error message was presented (“Zu schnell” [Too fast], “Zu langsam” [Too slow], “Falsche Taste” [Wrong button], or “Festes Muster” [Fixed pattern]) and the button press was not followed by a sound.

Each condition was preceded by a detailed explanation including the task and the relation between button presses and sounds and a training block. Blocks were separated by short breaks. The order of conditions and the assignment of frequent and rare button to the participants’ left and right hand was counterbalanced across participants.

### Stimuli and apparatus

Participants were comfortably seated in a dimly lit, sound attenuated, and electrically shielded booth. They held a response pad with buttons under the index fingers of their left and right hand. Sounds consisted of triangle waves (containing only odd harmonics with an amplitude ratio proportional to the inverse square of the harmonic number) with a frequency of 352 Hz (low sound; *F*^4^) or 440 Hz (high sound; *A*^4^) with a duration of 200 ms including 5 ms rise and 5 ms fall time (raised cosine window). Sounds were presented 400 ms after a button press via headphones (Sennheiser HD 25) at an intensity of 65 dB SPL. An LCD-computer screen was placed about 130 cm in front of the participants’ eyes so that visual stimuli appeared slightly below the horizontal line of sight. The visual display consisting of white digits on black background was separated into two rows. In the first row either the interval between the last two button presses in ms or an error message was displayed in case the button was pressed too fast or too slow or a wrong button was pressed. In the second row the number of remaining button presses per button per block and the ratio of remaining rare to frequent button presses in percent was displayed. The numbers of remaining button presses were presented spatially corresponding to the buttons. The visual display was updated immediately after a button press and subtended a visual angle of 2.5° × 0.75° (W × H).

### Data recording

The EEG was recorded with Ag-AgCl electrodes from 27 standard positions of the extended 10-20-system (Fp1, Fp2, F7, F3, Fz, F4, F8, FC5, FC1, FC2, FC6, T7, C3, Cz, C4, T8, CP5, CP1, CP2, CP6, P7, P3, Pz, P4, P8, O1, and O2) and from the left and right mastoids (M1 and M2). All electrodes were referenced to the tip of the nose. The vertical electrooculogram (EOG) was recorded between Fp1 and an infraorbitally placed electrode and the horizontal EOG between the outer canthi of the two eyes. Impedances of all electrodes were kept below 10 kΩ. EEG and EOG were filtered online with a bandpass of 0.1–250 Hz and sampled with a digitization rate of 500 Hz (BrainAmp, Brain Products, Gilching, Germany). Time was recorded for each button press.

### Data analysis

The EEG data were pre-processed using EEGLAB (Delorme and Makeig, 2004). Data were filtered offline with a 48 Hz low-pass filter (415-point Hamming-windowed sinc FIR filter, transition band width = 4 Hz; Widmann et al., 2015) and a 0.1 Hz high-pass filter (8,251-point Hamming-windowed sinc FIR filter, transition band width = 0.2 Hz). Data were divided into epochs of 0.6 s time-locked to sound onset, including a pre-stimulus baseline of 0.1 s. Only trials where the previous trial consisted of a frequent button press followed by a standard sound were included in the analysis. We excluded all epochs with signals exceeding peak-to-peak amplitudes of 500 μV at any electrode (to remove large non-stereotypical artifacts but to keep stereotypical artifacts as blinks and eye-movements to be later removed using ICA). Channels (except Fp1, Fp2, M1, M2, or EOG channels) were excluded if they had a robust z score of the robust standard deviation greater than 3 (Bigdely-Shamlo et al., 2015; a single channel in two participants). Artifacts were corrected with an independent component analysis (ICA), using the AMICA algorithm (Delorme et al., 2012). For the ICA, the 48 Hz low-pass filtered data were filtered with a 1 Hz high-pass filter (827-point Hamming-windowed sinc FIR filter, transition band width = 2 Hz), and divided into epochs of 0.6 s (removing the same channels and trials as in the previous step) but not baseline-corrected (Groppe et al., 2009). We then applied the obtained de-mixing matrix to the 0.1-48 Hz filtered data (Klug and Gramann, 2021). Artifact ICs were detected with support of the ICLabel plugin (Pion-Tonachini et al., 2019). All eye-movement (horizontal and vertical movements of the corneo-retinal dipoles and pre-saccadic spike potentials; Plöchl et al., 2012) and blink related artifact IC activity was subtracted from the data. On average, 4.5 ICs were removed from the data per participant (*Mdn* = 4; *min* = 4; *max* = 6). Bad channels were interpolated using spherical spline interpolation. Data were baseline corrected using the 0.1 s window before stimulus presentation. Finally, epochs with signals exceeding peak-to-peak amplitudes of 150 μV were excluded. Individual average ERPs were computed per participant for mispredicted (*mean/min/max N* of included trials per participant 136.9/127/144), predicted (141.7/129/165), and unpredictable deviants (247.9/239/253), and frequent (629.1/607/646) and rare button standards (104.4/89/133). As previously reported by Rinne et al. (2001) we also observed slightly different ERPs to standard sounds following a frequent button press and standard sounds following a rare button press in the unpredictable condition. To exclude differences between mispredicted and predicted deviants being based on the different frequency of the related button press, difference waves were calculated subtracting the ERPs to rare button standards from the ERPs to predicted deviants and the ERPs to frequent button standards from the ERPs to mispredicted deviants (as done similarly by Rinne et al., 2001). Grand average waveforms were computed from the individual average ERPs per stimulus type.

### Statistical analysis

There is no final consensus on the nomenclature for N1, MMN and P3a in the field. This is because each of these three components presumably consists of several subcomponents, which cannot easily be disentangled from each other, and because the components overlap in time (i.e., N1 with MMN, and MMN with P3a) with each other and also with other components (e.g., P2 and N2). In other words, the identification of ERP components in the measured ERPs is obscured because the measured ERPs are a mixture of latent underlying (sub-) components. Spatial and temporal overlap considerably biases the observed component peaks typically used to identify and label components (Scharf et al., 2022). Moreover, the practice of determining time windows for the respective components based on (peaks in) the observed ERP frequently suffers from the relatively arbitrary definition of time windows and double dipping (Kriegeskorte et al., 2009). Temporal PCA largely reduces these problems (e.g., Dien, 2012; Scharf et al., 2022). For that reason, we used temporal PCA to delineate the components in a straight-forward, data driven approach.

We conducted temporal principal component analysis (PCA) on the individual average ERP data of all channels and stimulus types using the tutorial code provided by Scharf et al. (2022). PCA was computed using Promax rotation (κ = 3) with a covariance relationship matrix (preferable over correlation matrix for ERP analyses as all sampling points are measured on the same scale; for discussion see, Dien et al., 2005; Scharf et al., 2022) and Kaiser weighting (to ensure that each variable has equal influence on the rotation process and therefore prevent that large factors dominate the results of the rotation step; for discussion see, Dien et al., 2005; Scharf et al., 2022). The number of components to be retained was determined using Horn’s parallel test. A total of 10 components was extracted. We focused our analyses on three components of interest, N1, MMN, and P3a.

Mean component scores were computed within frontal (FC5 and FC6; N1 and MMN), mastoidal (M1 and M2; N1 and MMN), and fronto-central (Fz, FC1, FC2, and Cz; P3a) regions of interest (ROI) centered on the observed spatial peaks across components (N1/MMN) and conditions. To obtain difference scores we subtracted component scores for frequent button standards from mispredicted and unpredictable deviants and rare button standards from predicted deviants (note that we only used standards from the unpredictable condition to correct for the confound introduced by different button press frequencies; cf. the last paragraph of the data analysis section above for a more detailed justification). For each component, stimulus type, and ROI, we computed one-sided Bayesian *t*-tests on the difference component scores (to verify that the components were elicited) and two-sided Bayesian *t*-tests for difference component scores of mispredicted vs. predicted deviants, mispredicted vs. unpredictable deviants, and predicted vs. unpredictable deviants (minus standards, respectively; to examine whether the components were modulated by condition) in R using the BayesFactor package (Morey and Rouder, 2021). The null hypothesis corresponded to a standardized effect size δ = 0, while the alternative hypothesis was defined as a Cauchy prior distribution centered around 0 with a scaling factor of *r* = 0.707 (the default “medium” effect size prior scaling). Data were interpreted as moderate evidence in favor of the alternative (or null) hypothesis if *BF*_10_ was larger than 3 (or lower than 0.33), or strong evidence if *BF*_10_ was larger than 10 (lower than 0.1). *BF*_10_ between 0.33 and 3 are considered as weak/anecdotal evidence (following Lee and Wagenmakers, 2013). In Table 1, we additionally report Cohen’s *d* effect sizes and frequentist *t*-tests for the tests of difference scores against baseline per component, stimulus type, and ROI.

**TABLE 1:** Deviant minus standard difference component scores for the PCA components N1, MMN, and P3a, effect sizes (Cohen’s *d*), and results of one-sided Bayesian and frequentist *t*-tests against baseline separately per deviant type and ROI.

Comp.	Deviant	ROI	Diff. score	*BF*_10_	*d*	*t*(14)	*p*
4/ΔN1	Mispredicted	Frontal	–0.61	84.75	–1.08	–4.20	<0.001
		Mastoidal	0.26	167.24	1.19	4.60	<0.001
	Predicted	Frontal	–0.56	974.65	–1.47	–5.70	<0.001
		Mastoidal	0.22	174.46	1.20	4.63	<0.001
	Unpredictable	Frontal	–0.63	3.95 × 10^4^	–2.15	–8.31	<0.001
		Mastoidal	0.27	3.63 × 10^3^	1.70	6.57	<0.001
3/MMN	Mispredicted	Frontal	–0.38	8.74	–0.73	–2.83	0.007
		Mastoidal	0.22	22.95	0.88	3.42	0.002
	Predicted	Frontal	0.21	0.12	0.36	1.41	0.910
		Mastoidal	–0.03	0.20	–0.10	–0.38	0.644
	Unpredictable	Frontal	–0.36	98.58	–1.11	–4.29	<0.001
		Mastoidal	0.19	38.35	0.96	3.72	0.001
1/P3a	Mispredicted	Fronto-central	1.18	2.41 × 10^3^	1.63	6.29	<0.001
	Predicted	Fronto-central	0.63	48.74	1.00	3.87	<0.001
	Unpredictable	Fronto-central	0.55	31.78	0.93	3.61	0.001

## Results

**FIGURE 2:**
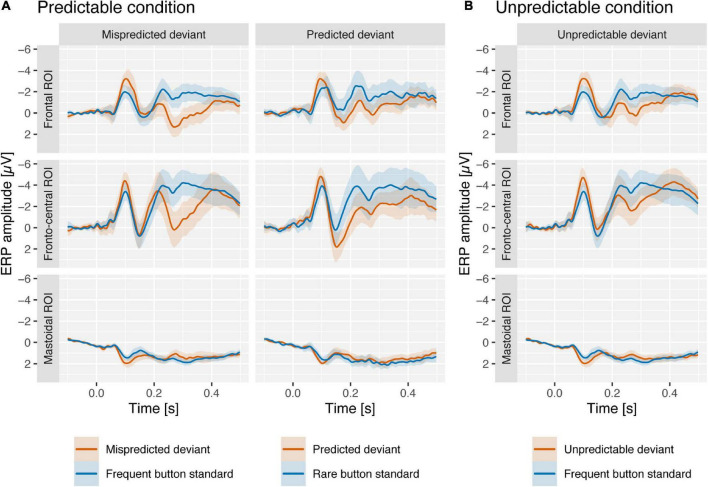
Grand-average ERPs at frontal ROI (FC5 and FC6), fronto-central ROI (Fz, FC1, FC2, and Cz), and mastoidal ROI (M1 and M2) from predictable **(A)** and unpredictable conditions **(B)** in response to mispredicted and predicted deviants (predictable condition; red) and unpredictable deviants (unpredictable condition; red). Deviants from both conditions are compared to frequent and rare button standards (blue) from the unpredictable condition (see “Materials and methods” section). Shaded areas reflect 95% confidence intervals. At around 100–150 ms ERPs are more negative for deviants than for standards at frontal and fronto-central regions, and more positive on mastoidal areas. At around 200–400 ms the ERPs for standards were more positive for deviants than for standards at fronto-central regions.

**FIGURE 3:**
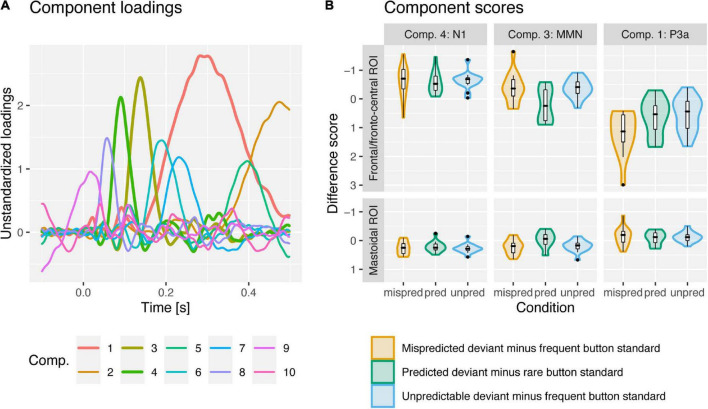
PCA component loadings **(A)** and violin and boxplots for deviant minus standard difference component scores **(B)** for mispredicted (orange; minus frequent button standards) and unpredictable deviants (blue; minus frequent button standards) and predicted deviants (green; minus rare button standards) for PCA components N1, MMN and P3a at frontal and mastoidal (N1 and MMN) and fronto-central ROIs (P3a). PCA components 4, 3, and 1 were associated with the N1, MMN and P3a ERP-components. N1 was enhanced (more negative at frontal, more positive at mastoidal electrode sites) for deviants compared to standards similarly in all conditions. MMN was observed for mispredicted and unpredictable deviants but abolished for predicted deviants. P3a was observed in all conditions but enhanced (more positive at fronto-central electrode sites) in response to mispredicted deviant compared to predicted and unpredictable deviants.

**FIGURE 4:**
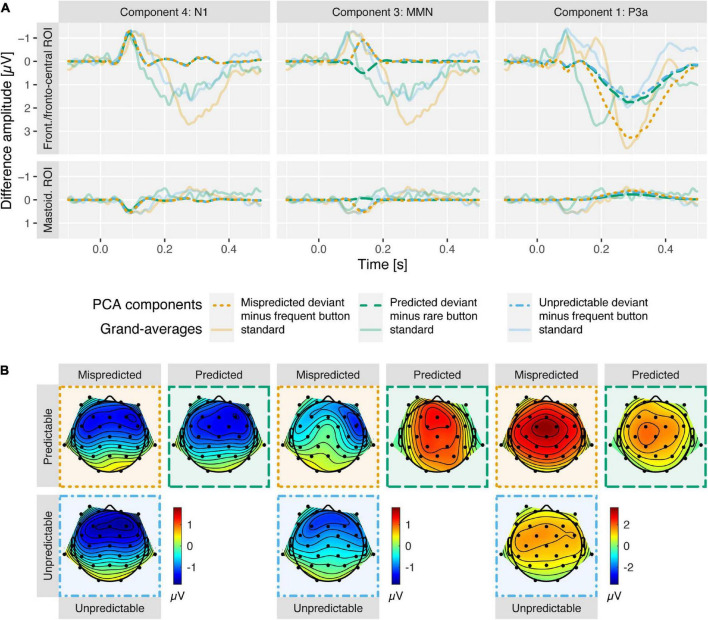
**(A)** Deviant minus standard difference waves, separately for N1, MMN, and P3a PCA components in columns one to three (opaque, dashed) at frontal and mastoidal (N1 and MMN) and fronto-central ROIs (P3a). For each column, the respective grand-average ERP differences waves are shown (transparent, solid) to enable a comparison between the time courses of the components scores and the ERPs. Note that N1 component traces overlap for all deviant types and MMN component traces overlap for mispredicted and unpredictable deviants. **(B)** Deviant minus standard difference topographies for N1 (90 ms), MMN (138 ms), and P3a (282 ms) PCA components at component peak latencies. In both panels mispredicted deviants (orange; minus frequent button standards) and predicted deviants (green; minus rare button standards) from predictable condition and unpredictable deviants (blue; minus frequent button standards) from unpredictable condition are displayed. N1 was enhanced (more negative at frontal, more positive at mastoidal electrode sites) for deviants compared to standards similarly in all conditions. MMN was observed for mispredicted and unpredictable deviants but abolished for predicted deviants. P3a was observed in all conditions but enhanced (more positive at fronto-central electrode sites) in response to mispredicted deviant compared to predicted and unpredictable deviants. Component score differences reveal topographies typical for N1, MMN and P3a.

In the following we will present results on the comparison of deviant vs. standard component scores per condition (the corresponding grand-average ERPs are displayed in Figure 2) and the comparison of deviant minus standard difference scores between conditions (the corresponding component loadings, difference scores and grand-average difference waves as well as topographies are displayed in Figures 3, 4, respectively) separately for the N1, MMN, and P3a PCA components.

### Component 4/ΔN1

N1 was reflected in PCA component 4 peaking 90 ms after stimulus onset. The data provided strong evidence for enhanced N1 component amplitudes at frontal (more negative) and mastoidal electrode locations (more positive) in response to all deviant types compared to standards (ΔN1; all *BF*_10_ > 80). The data provided moderate evidence against a difference of ΔN1 amplitudes between mispredicted and predicted deviants [frontal ROI: *BF*_10_ = 0.28, *d* = −0.09, *t*(14) = −0.33, *p* = 0.744; mastoidal ROI: *BF*_10_ = 0.33, *d* = 0.19, *t*(14) = 0.74, *p* = 0.471] and moderate evidence against a difference of ΔN1 amplitudes between mispredicted and unpredictable deviants [frontal ROI: *BF*_10_ = 0.27, *d* = 0.06, *t*(14) = 0.22, *p* = 0.83; mastoidal ROI: *BF*_10_ = 0.27, *d* = −0.07, *t*(14) = −0.26, *p* = 0.799] as well as moderate evidence against a difference between predicted and unpredictable deviants at frontal electrode locations and inconclusive evidence at mastoidal electrode locations [frontal ROI: *BF*_10_ = 0.32, *d* = 0.18, *t*(14) = 0.69, *p* = 0.499; mastoidal ROI: *BF*_10_ = 0.94, *d* = −0.46, *t*(14) = −1.79, *p* = 0.096].

### Component 3/mismatch negativity

Mismatch negativity was reflected in PCA component 3 peaking 138 ms after stimulus onset. The data provided moderate to strong evidence for the elicitation of a frontal MMN component inverting polarity over mastoidal electrode locations for mispredicted and unpredictable deviants (all *BF*_10_ > 8) and moderate to strong evidence against the elicitation of a MMN component for predicted deviants (all *BF*_10_ < 0.25). The data provide moderate to strong evidence for a difference of MMN amplitudes between mispredicted and predicted deviants [frontal ROI: *BF*_10_ = 3.05, *d* = −0.67, *t*(14) = −2.6, *p* = 0.021; mastoidal ROI: *BF*_10_ = 14.18, *d* = 0.91, *t*(14) = 3.54, *p* = 0.003] and moderate evidence against a difference of MMN amplitudes between mispredicted and unpredictable deviants [frontal ROI: *BF*_10_ = 0.27, *d* = −0.04, *t*(14) = −0.16, *p* = 0.876; mastoidal ROI: *BF*_10_ = 0.31, *d* = 0.16, *t*(14) = 0.63, *p* = 0.537] as well as strong evidence for a difference between predicted and unpredictable deviants [frontal ROI: *BF*_10_ = 10.54, *d* = 0.87, *t*(14) = 3.36, *p* = 0.005; mastoidal ROI: *BF*_10_ = 29.46, *d* = −1.03, *t*(14) = −3.98, *p* = 0.001].

### Component 1/P3a

The P3a was reflected in PCA component 1 peaking 282 ms after stimulus onset. The data provided strong evidence for the elicitation of a fronto-central P3a component for all deviant types (all *BF*_10_ > 30). The data provide anecdotal/weak to moderate evidence for a difference of P3a amplitudes between mispredicted and predicted deviants [fronto-central ROI: *BF*_10_ = 2.72, *d* = 0.65, *t*(14) = 2.53, *p* = 0.024], strong evidence for a difference of P3a amplitudes between mispredicted and unpredictable deviants [fronto-central ROI: *BF*_10_ = 18.89, *d* = 0.96, *t*(14) = 3.71, *p* = 0.002], and moderate evidence against a difference between predicted and unpredictable deviants [fronto-central ROI: *BF*_10_ = 0.28, *d* = 0.1, *t*(14) = 0.38, *p* = 0.709].

## Discussion

The present study aimed at determining the effects of action-effect intention on auditory oddball processing. Active listeners produced standard and deviant (oddball) sounds by pressing one of two buttons, one button frequently and the other button rarely. In an unpredictable condition the type of button to be pressed (frequent and rare) was unrelated to the type of sound produced (standard and deviant); standard and deviant sounds were “unpredictable” for the participant. In a predictable condition, the frequent button produced a standard sound and the rare button a deviant sound in most trials. Participants were asked to generate standards by pressing the one button frequently and deviants by pressing the other button rarely. Most deviants were correctly “predicted”; importantly however, occasionally a button press for a standard triggered a (“mispredicted”) deviant. It turned out that (1) the deviance-specific N1 enhancements were highly similar between the three different deviant types (unpredictable, correctly predicted, and mispredicted), (2) that MMN was highly similar for mispredicted and unpredictable deviants, but no MMN was elicited for predicted deviants, (3) that predicted and unpredictable deviants elicited similar P3a, whereas the P3a for mispredicted deviants was enlarged. Thus, the system underlying the N1 deviance processing was not modulated depending on whether an intended action effect did or did not occur. The MMN-system was modulated if the action intention was confirmed (MMN reduced or abolished for predicted deviants) but not if the action intention was violated. Mispredicted deviants violating both auditory regularity and action intention did not elicit an enhanced MMN compared to unpredictable deviants (violating auditory regularity only). In contrast, the P3a-system was affected if the action intention was violated (P3a enhanced for mispredicted deviants) but not if it was confirmed (P3 was not reduced or abolished for predicted deviants).

### No impact of action intention on deviance-specific N1 enhancement

Many studies showed that the auditory N1 is attenuated for self-generated sounds supporting motor-to-sensory forward-modeling accounts of sound processing (for reviews see, Horvath, 2015; Schröger et al., 2015). If the N1 *per se* can be modulated by intentional action, it seems reasonable to assume that also the deviance-specific enhancement of the N1 can be attenuated for intended action effects. Moreover, according to predictive coding theory (Friston et al., 2010; Clark, 2013) such an effect would be expected. On the other hand, according to the adaptation model by May (2021) such an effect is not (necessarily) to be expected as the N1 enhancement to deviants can be explained by bottom-up driven short-term synaptic depression of neurons in auditory cortex, which does not involve top-down processing. Indeed, our study revealed deviance-specific N1 enhancement at around 90 ms which was highly similar for unpredictable deviants, correctly predicted deviants, and mispredicted deviants. That is, the N1 enhancement to violations of an auditory-regularity was not influenced by the intention-based sound predictions.

Complementary evidence for the functional independence of oddball processing from intentional action at the N1-level has been reported by Korka et al. (2019), who found that sounds that violated the intention-based prediction did not cause an N1 enhancement (but MMN and P3a, see below). Correspondingly, Darriba et al. (2021) did not find an N1 effect in this time-range when an intention-based prediction was violated. Together, these studies suggest (though from different angles) that the N1-system is sensitive to auditory regularity violations, but apparently not to violations of intention-based predictions. If the system underlying N1 generation is not sensitive to violations of intention-based predictions, it seems possible that the N1 enhancement for violations of an auditory regularity is also not a direct expression of prediction error processing and may possibly better be explained more parsimoniously, without referring to prediction violation (May, 2021). It should be noted that adaptation (in the sense of repetition suppression) presumably underlying the auditory oddball N1 effect has been explained in terms of more precise, optimized predictions about sensory inputs (Auksztulewicz and Friston, 2016). In the light of this theory, it is somewhat surprising that the violation of an expected action effect did not matter for the oddball N1 effect but confirms the functional separation of N1 vs. MMN reflecting adaptation-driven vs. genuine prediction-driven deviance processing (Quiroga-Martinez et al., 2020; Schröger and Roeber, 2021).

### Strong impact of action intention on mismatch negativity when the action intention is confirmed: Top-down influence on mismatch negativity

The finding that MMN was elicited for unpredictable deviants and for mispredicted deviants but not for predicted deviants shows that MMN is modulated by the top-down influence of the action intention prediction. Even though the deviant violated an auditory regularity, it did not elicit an MMN when the brain was informed by the intention-based prediction that a deviant sound will occur. At a first glance, the present results seem to be at odds with previous research suggesting that MMN cannot be modulated in a top-down manner by preceding visual or by action effect information. Previous studies (Ritter et al., 1999; Sussman et al., 2003) found no top-down modulation of MMN with visual cues informing about forthcoming deviants (though P3a was affected). This is evidence that this kind of visual cuing has no impact on the auditory regularity-based deviance detection system. On the other hand, the present results were to be expected on the basis of recent research showing that the violation of predictive information provided from non-auditory processing modules (vision and action intention) may elicit MMN in the absence of an auditory-regularity: First, sounds violating a prediction which has been induced by visual symbolic information (i.e., music notation) elicit a so-called visuo-auditory incongruency response (IR; e.g., Widmann et al., 2004; Aoyama et al., 2006). The IR shares essential features of MMN, namely, latency and generators in supratemporal areas (Pieszek et al., 2013), so that it might be interpreted as a top-down, non-oddball variant of MMN. Second, MMN can be elicited by the violation of an intention-based prediction for an upcoming sound, when there is no auditory regularity (Korka et al., 2019). If MMN can be elicited in the absence of an auditory regularity via predictive information delivered top-down from non-auditory modules, it seems likely that MMN for the violation of an auditory regularity can also be modulated by top-down predictive information of intentional action. Taken together, the finding that MMN can be elicited by sounds violating a visual-based prediction (Widmann et al., 2004) or an intended action effect only (Korka et al., 2019) and the finding that action intention can abolish the MMN for the violation of an auditory regularity (present study) reveal that intentional action exerts a strong impact on the MMN system: it can turn the MMN system on (Korka et al., 2019) or off (present study). In sum, the present finding is consistent with predictive coding theory, where the action system is attributed a privileged role in changing sensations and overriding sensory predictions (e.g., Friston et al., 2010; Brown et al., 2013).

### No impact of action intention on mismatch negativity when the action intention is violated: No mismatch negativity amplitude increase for concurrent violations of regularity and intention

The present experiment was designed to enable the concurrent establishment of two generative models, the one considering the auditory regularity, the other considering the intended action effect. This poses the question what happens if both models either generate contradictory or congruent predictions about the forthcoming sound: In the case of mispredicted deviants the predictions are congruent, in the case of predicted deviants they are contradictory. Mispredicted deviants (violating the auditory regularity and the intention-based prediction) should elicit larger MMN than unpredictable deviants (only violating the auditory regularity). This was not the case. MMN (Paavilainen et al., 2001; Wolff and Schröger, 2001), IR and MMN (Pieszek et al., 2013), and N1b (Darriba et al., 2021) studies yielded enlarged MMN, IR, and N1b, respectively, when two regularity predictions were violated in parallel as compared to when only one regularity prediction was violated. The absence of an MMN increase for regularity plus action intention deviants relative to single, regularity only deviants in the present study points to the special role of action intention as outlined in the predictive coding theory (Brown et al., 2013). At a first glance, the additivity of prediction violation effects on the N1b reported by Darriba et al. (2021) for violations of the auditory regularity (established by the learned sound pattern) and the action intention (referring to the same sound feature) seem to contradict this interpretation. We propose that the difference in the results between the Darriba et al. (2021) and the present study are due to two differences in the experimental designs. (1) In Darriba et al. (2021) action intention was established before sensory regularity. The task cue was presented before the sound pattern. In the present study sensory regularity was established before action intention. (2) In Darriba et al. (2021) the sensory regularity was established independently of action intention; auditory regularity and action intention were manipulated orthogonally. Thus, prioritizing one over the other would not have resulted in better predictions. However, in the predictable condition in the present study, sensory regularity and action intention were correlated and mutually dependent. Selecting the rare button predicted the deviant sound action effect with high probability and therefore presumably gave rise to an adjustment of the regularity-based generative model. Prioritizing action intention overall resulted in better predictions. This interpretation is compatible with results demonstrating stronger expectations due to the intention to produce a specific auditory effect relative to the expectation due to stimulus-driven expectancy which has been reported during music performance (Maidhof et al., 2010).

In the context of auditory scene analysis it has been claimed that several auditory regularity-based predictive representations can coexist (Mill et al., 2013; Schröger et al., 2014; Szabo et al., 2016). This corresponds to the situation of parallel processing of the violation of concurrent regularities underlying MMN and IR-additivity and N1b-additivity. However, according to a computational model of auditory scene analysis these concurrent predictive representations compete with each other when it comes to the next level of processing, which is conscious perception in the context of auditory scene analysis (e.g., Mill et al., 2013). In the light of this model, it seems possible that a competition between the two predictive regularities happened in the present study and that intention-based violation detection processing took over, while the auditory regularity-based violation detection processing was attenuated. In other words, these two processing systems may not be organized in a modular fashion in a situation where the intention-based prediction system is in charge. From a more general view, this perspective is in line with studies showing that context is highly relevant for modulations of early auditory processing (e.g., Dercksen et al., 2021); and, vice-versa, the execution of a simple action (e.g., a right button-press) depends on the specific context, for example, whether the button-press denotes a “yes” or a “no”-answer (Aberbach-Goodman et al., 2022).

In view of the present and previous results we suggest that at the MMN-level (1) several predictions relating to the same or different features of a sound can be maintained and mismatched concurrently (MMN-additivity). If (2) congruent predictions result from different generative models (bottom-up extracted auditory regularity, top-down visual-auditory predictive association) functional independence (evidence accumulation) for prediction violations is achieved (IR/MMN/N1b-additivity). Importantly, (3) intention-based predictions may overwrite the auditory regularity-based prediction depending on context (note that this has been demonstrated also for the case of congruent predictions showing no additivity; Korka et al., 2019). Suggestion (3) is consistent with predictive coding theory according to which the prediction error is weighted by the confidence in the sensory data (Friston, 2005; Brown et al., 2013; Clark, 2013). Confidence (precision) can be modulated by attention (Feldman and Friston, 2010) and by active inference induced by actions (Friston et al., 2010; Brown et al., 2013). Active inference is involved in our task, where participants produced standards and deviants by intentional actions. Considering that “under active inference, perception tries to explain away prediction errors by changing predictions” (Friston et al., 2010, p. 235) the observed primacy of the intention-based prediction over the auditory regularity-based prediction at the MMN-level is to be expected according to the predictive coding theory. Our result is also supportive of Clark’s (2015) provocative claim that “motor control is just more top-down sensory prediction”.

### Impact of action-intention on P3a when action-intention is violated, but not when it is confirmed

All three deviant types elicited a P3a. While unpredictable and predicted deviants elicited P3a of comparable amplitude, the P3a for mispredicted deviants was enlarged. The P3a increase for deviants that violated an auditory-regularity and an action-effect intention replicates previous findings (Nittono, 2006; Waszak and Herwig, 2007; Herwig and Waszak, 2009; Knolle et al., 2013; Darriba et al., 2021). Waszak and Herwig (2007) interpret the P3a increase to deviants when the action intention actually predicted a standard as an increase in the orienting response (Waszak and Herwig, 2007). Consistently, Darriba et al. (2021) argued that the auditory regularity-based and the intention-based predictions were not integrated but remained independent. Our results are compatible with this interpretation.

Interestingly, the P3a elicited by a sound violating an auditory regularity does not differ between predicted and unpredictable deviants. Metaphorically spoken, although the P3a-system does care when the action intention is violated (replicating previous findings, see above), it does not care when it conforms to the action intention (that is, it is enhanced for mispredicted but not reduced for correctly predicted deviants). On the one hand, this is not what one would expect based on the MMN results, characterized by an absence of MMN for predicted deviants. On the other hand, this result is compatible with the idea that the P3a-system evaluates stimuli with respect to their ‘significance’ by combining the stimulus information with its relevance within a wider context (here, additively integrating violations of both sensory regularity and action intention; Horvath et al., 2008; Wetzel et al., 2013) eventually activating the organism’s resources for action (Nieuwenhuis et al., 2011). Thus, our results are compatible with the notion that prediction error increases and adaptation decreases with higher level within the cortical hierarchy obtained from human imaging studies (Schlossmacher et al., 2022) and electrophysiological animal (Parras et al., 2021) studies. However, the present results also reveal that the P3a-system still considers the information about a deviancy from the auditory regularity (which has been assessed already at the N1 level).

### Limitations

Amongst the limitations of the present study is that we cannot be sure about the divergence of the MMN results between the Rinne et al. (2001) and the present study, with regular MMN for predicted deviants in the Rinne study but NO MMN for predicted deviants in the present study. We suspect that it is the difference between the instructions in these two studies resulting in quite contradictory effects. While in the Rinne study participants were instructed to press buttons, they were instructed to produce sounds in the present study. In the context of ideomotor theory, it has been argued that actions are only selected with respect to their anticipated sensory effects in a so-called “intention-based action mode” (Herwig and Waszak, 2009). If one assumes that the action performed by the participants were not sufficiently strongly associated to its effect, and–consequently–did not give rise to respective predictions for the forthcoming sounds, a modulation of the MMN is not necessarily to be expected in the study by Rinne et al. (2001). Such striking effects of a (presumably) minor change in instruction has, for example, be shown on the Simon effect, a stimulus-response spatial compatibility effect (Hommel, 1993). In this study by Hommel, it turned out that when participants intended to switch on a (left or right lateralized) light, rather than to press a (left or right) button as response to a lateralized sound, the Simon effect inverts. Though we believe that the difference in instruction is the cause for the striking difference in MMN results, there are two further differences between the studies, which could possibly play a role. In the Rinne et al. (2001) study, the auditory regularity-based and the action intention-based predictions were fully predictable. That is, unlike to the present study, the contingency relations in the Rinne study did not promote the need to monitor the outcome of the actions. Finally, the Rinne study utilized duration deviants, whereas the present study used pitch deviants. Also, this difference could, in principle, be responsible for the difference in MMN results.

Another limitation of the present study is that we cannot fully exclude that participants may occasionally have thought they made a mistake when an unexpected tone occurred. This, in turn, might have resulted in motor error-related ERPs (e.g., ERN). We have intentionally tried to prevent this by the instructing participants not to care about rare, unexpected sounds. Also, when performing this task, the occurrence of a mispredicted tone does not “feel like” that one has committed a motor error, but it rather sounds like an auditory deviant. Also, the topography of the N1 and MMN effects, with polarity reversal at mastoidal leads (Figure 4) pointing at generators in supratemporal areas argues against the possibility that we might misinterpret an ERN as an oddball-N1 or MMN. Anyway, we see no way to disentangle the two cases where participants did not think that they made a (motoric) mistake but noticed that an unpredicted sound occurred versus where participants noted the unexpected sound and ascribed it to a motoric mistake of their own. Thus, we decided to avoid speculations on possible (and interesting) relations between the present auditory oddball ERP effects and motor error-related ERPs in the present paper.

## Conclusion

In sum, the impact of the violation (or confirmation) of an intention-based prediction on auditory-regularity-based deviant processing is (at least) threefold. (1) The pattern of results for the early-level (N1) processing is compatible with stimulus-driven neural adaptation mechanisms, which can be explained without referring to predictive processing (May, 2021), but which is also compatible with a predictive coding account (Auksztulewicz and Friston, 2016). (2) The pattern of results for the intermediate level (MMN) processing is supportive of generalized predictive coding theory that includes action (Friston et al., 2010; Clark, 2013). Although stimulus-driven and intention-driven effects take place at this level, intention-based predictive processing may be prioritized over the stimulus-driven effects depending on context. (3) Results for the late-level (P3a) processing support the idea that the P3a indicates an overall accumulation process considering the available information for deviants detected at the earlier levels (Winkler and Schröger, 2015).

## Data availability statement

The raw data supporting the conclusions of this article will be made available by the authors, without undue reservation.

## Ethics statement

Ethical review and approval was not required for the study on human participants in accordance with the local legislation and institutional requirements. The patients/participants provided their written informed consent to participate in this study.

## Author contributions

AW and ES equally contributed to conception and design of the study, writing the first draft and manuscript revision, and read and approved the submitted version. AW implemented the study and performed data and statistical analysis. Both authors contributed to the article and approved the submitted version.

## Footnotes

1
https://www.dgps.de/die-dgps/aufgaben-und-ziele/berufsethische-richtlinien/
